# SSR markers for *Filago* subg. *Filago* (Gnaphalieae: Asteraceae) and cross‐amplification in three other subgenera

**DOI:** 10.1002/aps3.1171

**Published:** 2018-08-21

**Authors:** David Gutiérrez‐Larruscain, Teresa Malvar Ferreras, M. Montserrat Martínez‐Ortega, Enrique Rico, Santiago Andrés‐Sánchez

**Affiliations:** ^1^ Departamento de Botánica y Fisiología Vegetal Facultad de Biología University of Salamanca Licenciado Méndez Nieto Ave. E‐37007 Salamanca Spain; ^2^ Biobanco de ADN Vegetal University of Salamanca Edificio I+D+i, Espejo St. E‐37007 Salamanca Spain; ^3^ Departamento de Didáctica de las Matemáticas y de las Ciencias Experimentales Facultad de Educación University of Salamanca Canalejas Ave. E‐37008 Salamanca Spain

**Keywords:** Asteraceae, *Evax*, *Filago*, microsatellites

## Abstract

**Premise of the Study:**

Microsatellite primers were developed for the first time in the genus *Filago* (Gnaphalieae: Asteraceae). These markers will facilitate low‐scale phylogenetic, phylogeographic, and population genetic studies within the genus *Filago*.

**Methods and Results:**

Ten pairs of polymorphic microsatellite primers (as well as five pairs of monomorphic primers) were identified and optimized on two species of *Filago* (*F. gaditana* and *F. carpetana*) using a microsatellite‐enrichment library method and 454 GS‐FLX technique. The polymorphic primers amplified tri‐ to hexanucleotide repeats and showed one to six alleles per locus for both species. Transferability was performed in 29 samples corresponding to nine representative species of *Filago*.

**Conclusions:**

The results indicate the utility of the newly developed markers, which will be useful to delve into the phylogenetic relationships among the taxa within *Filago*. These microsatellites will enable studies of phylogeographic, reproductive, and genetic variation.

The genus *Filago* Loefl. ex L. (Asteraceae: Gnaphalieae) comprises ca. 45 species grouped into four subgenera (Galbany‐Cassals et al., 2010; Andrés‐Sánchez et al., 2011). It is composed of annual ephemeral plants that grow in open, often disturbed, dry habitats, but some species are stenoic and ecologically restricted to particular habitats such as salt marshes or small snowbeds at high altitudes. Some of the species are considered weeds (Carretero, 2004; Randall, 2007) and others are listed on either national or regional catalogs of endangered plants (Barreno et al., 1985; Moreno, 2008) due to their narrow distribution areas (Andrés‐Sánchez et al., 2013). Eight of the species traditionally included within the genus *Evax* Gaertn. represent a monophyletic group (hereafter named the *Evax* group) currently placed in *Filago* subg. *Filago* (Andrés‐Sánchez et al., 2015).

To develop microsatellite markers for *Filago*, we chose a small subclade within the *Evax* group, which includes *F. carpetana* (Lange) Chrtek & Holub and *F. gaditana* (Pau) Andrés‐Sánchez & Galbany. These species are characterized by disjunct distributions, restricted to the Iberian Peninsula and France, and to the Iberian Peninsula and northwestern Morocco, respectively. Considering that autogamy s.l. (i.e., including geitonogamy) has been frequently related to long‐distance dispersal and with the colonization of new areas (Obbard et al., 2006), these species represent a suitable model to develop biogeographic studies on annual plants in the western Mediterranean region (e.g., long‐distance dispersal events related to autogamy, effects of the absence of evident dispersal mechanisms). The development of codominant markers will allow for the collection of data on the prevalence of autogamy in the populations of *Filago*, as well as on gene flow.

Hypervariable genetic markers are also needed to overcome problems related to the scant variability detected in nuclear and plastid DNA markers (Galbany‐Cassals et al., 2010; Andrés‐Sánchez et al., 2015). The transferability of loci to other species would allow for the development of studies aimed to understand the phylogenetic relationships within the genus *Filago*.

## METHODS AND RESULTS

### Microsatellite development

Silica gel–dried leaf material from 11 samples of *F. carpetana* and *F. gaditana* were used for the preparation of the microsatellite library (Appendix 1). Total DNA was extracted following the cetyltrimethylammonium bromide (CTAB) extraction protocol (Doyle and Doyle, 1987) with minor modifications. The library was prepared by Genoscreen (Lille, France) and sequenced using a 454 GS‐FLX (Roche Diagnostics, Meylan, France) high‐throughput DNA sequencer (Malausa et al., 2011). The DNA was fragmented and enriched TG, TC, AAC, AAG, AGG, ACG, ACAT, and ACTC motifs. A total of 25,692 sequence reads were obtained (data available from the Dryad Digital Repository: https://doi.org/10.5061/dryad.94g0tc5; Gutiérrez‐Larruscain et al., 2018). These sequences were analyzed with the software QDD2 (Meglécz et al., 2014) revealing 3160 sequence reads with microsatellite motifs. From 63 primer pairs with A design (Meglécz et al., 2014), a total of 30 with low penalty values, different lengths, and repeat motifs were selected. These primers were ordered (Eurofins, Ebersberg, Germany) to check the variability of these loci in two samples of *F. carpetana* and two of *F. gaditana*. PCRs were performed in 12.5‐μL volume reactions, which contained 45.5 ng of DNA template, 1.25 μL of 1× PCR buffer (Biotools, Madrid, Spain), 1.5 mM MgCl_2_ (Biotools), 0.2 mM of each dNTP (Life Technologies, Carlsbad, California, USA), 0.33 mM of each primer, and 0.5 units of DNA Polymerase (Biotools). PCR was performed in an Eppendorf thermocycler (Mastercycler ProS; Eppendorf, Hamburg, Germany), using the following conditions: an initial denaturation step at 94°C for 2 min; followed by 30 cycles of 1 min at 94°C denaturation, 45 s at 55°C annealing, and 1 min 30 s at 72°C extension; with a final extension of 10 min at 72°C. PCR products were envisioned on a 2.5% agarose gel and sent to Macrogen Europe sequencing service (Amsterdam, The Netherlands). The obtained sequences were examined to assess homology and correct amplification. Fifteen primers were selected and tested in three populations of *F. carpetana* and three populations of *F. gaditana* (Appendix 1; primers discarded and reasons for discarding are shown in Appendix 2). The sequence‐specific forward primers were marked using the universal primer M13(–21) 5′‐TGTAAAACGACGGCCAGT‐3′ (Schuelke, 2000) labeled with 5‐FAM, VIC, NED, or PET fluorescent dyes (Table 1) (Life Technologies). The composition of the PCR mastermix for populations SA865 and SA1109 was as described above, except for the fluorescent‐labeled reverse primer (0.8 mM) and the forward primer (0.2 mM). For populations DP2044, DP2040, DG1052, and SA1218, PCRs were performed in 15‐μL volume reactions, which contained 45.5 ng of DNA, 3 μL of 1× Green GoTaq buffer (Promega Corporation, Madison, Wisconsin, USA), 0.2 mM of each dNTP, 0.04 mM of forward primer, 0.16 mM of fluorescent‐labeled reverse primer, 0.75 units of GoTaq polymerase (Promega Corporation), 0.7 μL of dimethyl sulfoxide (DMSO; Fisher Scientific, Hampton, New Hampshire, USA), and 0.3 μL of bovine serum albumin (BSA) 1 mg/mL (New England Biolabs, Ipswich, Massachusetts, USA). Regarding PCR conditions, annealing temperature was changed to 1 min at 52°C and extension temperature was changed to 50 s at 72°C for the first 30 cycles. The annealing temperature of the last 10 cycles was increased to 53°C. For the markers mf14 and mf25, the denaturation temperature was decreased to 83°C, and the annealing temperature was 52°C for 1 min for 35 cycles. The PCR products were run on an ABI 3730 Capillary Sequencer (Life Technologies) using GeneScan 500 LIZ Size Standard (Life Technologies). Electropherograms were analyzed with GeneMarker AFLP/Genotyping Software version 1.8 (SoftGenetics, State College, Pennsylvania, USA). Seven primers were discarded because they were monomorphic for all species analyzed or unspecific. In the cases that the expected sizes of the alleles were different than those obtained, the individuals were sequenced in order to identify indel presence.

**Table 1:** Characteristics of 15 microsatellites amplified in *Filago*.^a^

Locus	Primer sequences (5′–3′)	Fluorescent dye	Repeat motif	Allele size range (bp)^b^	*T*_a_ (°C)	*T*_d_ (°C)	GenBank accession no.
mf1^c^	F: ACCCACGAGTTAATATGCCG	FAM	(AAC)_5_	91	52–53	94	KY792553
	R: TACTTAACCGGTCCCAGGC						
mf3^c^	F: TGGATAAGGGATTTAGCATTGG	VIC	(ACC)_5_	121	52–53	94	KY792554
	R: CGGTCGTTTGCTCGTTATCT						
mf5	F: GCAGAATCACATTCAACTCACG	NED	(AGAT)_5_	131–146	52–53	94	KY792555
	R: ATGAGCTAGAGAAATAACTGATGTT						
mf7^c^	F: TACCATTTGACCATGCGTTT	PET	(AAG)_5_	131	52–53	94	KY792556
	R: CTTTCTTTGTGTTGTTCCTTCG						
mf8	F: TTCGGTTACTGTTGCATCTAGG	FAM	(AAG)_6_	150–171	52–53	94	KY792557
	R: ATTAACCGGAGGAGTTTGGA						
mf9	F: ACTGAAGCGCGAACAATCTC	VIC	(AAG)_6_	154–169	52–53	94	KY792558
	R: CCACTACAGATGACTCGGCA						
mf10	F: TATGTATCACGCGCCTATGG	NED	(AAGGTC)_7_	137–156	52–53	94	KY792559
	R: CACTGTAAAGATCCGACGGC						
mf12^c^	F: ATTGTTAGGGTTGGTGGTCG	PET	(ACC)_5_	144	52–53	94	KY792560
	R: CAAACATTCCTGGGTATGGG						
mf13	F: GACTTCAAATCTGGATGAATTT	FAM	(AAG)_8_	146–171	52–53	94	KY792561
	R: ACCATATGCACCGATTGATT						
mf14	F: CGACAGTAAATACTCATTGAACCA	VIC	(ACAT)_5_	161–181	52–53	83	KY792562
	R: GGTATCTTTCGTCATGTAACATTCA						
mf19	F: TTTCTGAACCAAGATCGGTATTC	FAM	(AGAT)_5_	244–256	52–53	94	KY792563
	R: TCGCTTTCTCCAGATCATCC						
mf20^c^	F: CAATCCCAAATCTGAAGCGT	FAM	(AAC)_5_	236	52–53	94	KY792564
	R: TTTGATTCTCCATGAGCAAGA						
mf25	F: ACACCACAAGGGCATGTGTA	FAM	(AAC)_5_	276–284	52–53	83	KY792565
	R: TCTTGTCACTAAGTAGTCCTATCGC						
mf26	F: AATATGTCACCGTCGGGTTC	VIC	(AAC)_5_	289–300	52–53	94	KY792566
	R: GTGTTCGGGTACAAATTCGG						
mf28	F: GGGAACTTGAACCATCATCC	VIC	(AAC)_6_	296–300	52–53	94	KY792567
	R: TCCATATTAGCTACACTCCCTTCA						

Note

*T*_a_ = optimal annealing temperature; *T*_d_ = optimal denaturation temperature.

a All values are based on 60 samples from *F. gaditana* and *F. carpetana*.

b Fragment size ranges do not include M13 tail.

c Monomorphic loci.

### Population genetic parameters in two species of *Filago*

The number of alleles per locus, levels of observed (*H*_o_) and expected heterozygosity (*H*_e_), significance of deviation from Hardy–Weinberg equilibrium (HWE; Table 2), and tests for linkage disequilibrium between markers were calculated using Arlequin version 3.5.1.2 (Excoffier and Lischer, 2010). The number of alleles ranged from one to six for both *F. gaditana* and *F. carpetana*. *H*_o_ and *H*_e_ values ranged from 0 to 1 and from 0.005 to 0.728, respectively, for all six populations. Deviation from HWE (*P* < 0.01) was detected in each population for all loci except for locus mf25. Discordant values of *H*_o_ and *H*_e_ and the subsequent deviation of HWE (except for locus mf25, which only was amplified for population SA1109) could be attributed to autogamy processes. Linkage disequilibrium was significant after Bonferroni correction for all pairwise comparisons except for those involving mf5 and mf14.

**Table 2:** Results of initial primer screening of 10 polymorphic loci in six populations^a^ corresponding to two species of *Filago*

Locus	*F. carpetana* DG1052 (*n* = 27)				*F. carpetana* SA1218 (*n* = 21)				*F. carpetana* SA1109 (*n* = 30)				*F. gaditana* SA865 (*n* = 30)				*F. gaditana* DP2044 (*n* = 27)				*F. gaditana* DP2040 (*n* = 28)			
	*A*	*H*_o_	*H*_e_	HWE^b^	*A*	*H*_o_	*H*_e_	HWE^b^	*A*	*H*_o_	*H*_e_	HWE^b^	*A*	*H*_o_	*H*_e_	HWE^b^	*A*	*H*_o_	*H*_e_	HWE^b^	*A*	*H*_o_	*H*_e_	HWE^b^
mf5	1	—	—	—	1	—	—	—	2	0.000	0.131	0.001***	3	0.000	0.508	0.000***	2	0.000	0.492	0.000***	1	—	—	—
mf8	2	1	0.509	0.000***	3	1	0.633	0.000***	6	1	0.687	0.000***	3	0.967	0.636	0.000***	2	1	0.509	0.000***	2	1	0.509	0.000***
mf9	1	—	—	—	1	—	—	—	3	0.033	0.501	0.000***	5	0.000	0.653	0.000***	3	0.037	0.174	0.001***	1	—	—	—
mf10	2	0.778	0.484	0.001***	4	0.619	0.728	0.000***	3	0.8667	0.005	0.000***	3	0.000	0.59	0.000***	1	—	—	—	1	—	—	—
mf13	2	0.963	0.509	0.000***	2	0.762	0.483	0.000***	4	0.8	0.561	0.000***	6	0.933	0.656	0.005**	2	0.037	0.465	0.000***	1	—	—	—
mf14	2	0.037	0.372	0.000***	3	0	0.621	0.000***	3	0.067	0.337	0.000***	3	0.000	0.472	0.000***	2	0.000	0.462	0.000***	1	—	—	—
mf19	2	0	0.391	0.000***	1	—	—	—	2	0.000	0.127	0.000***	2	0.000	0.452	0.000***	1	—	—	—	1	—	—	—
mf25	‡				‡				4	0.433	0.367	1.000^ns^	1	—	—	—	1	—	—	—	1	—	—	—
mf26	‡				‡				5	0.033	0.536	0.000***	2	0.000	0.127	0.001***	‡				‡			
mf28	2	1	0.508	0.000***	2	0.524	0.396	0.000***	3	0.000	0.513	0.000***	3	0.033	0.186	0.000***	2	1	0.509	0.000***	2	1	0.509	0.000***

Note

*— =* no population genetic analyses were performed for monomorphic loci; *A* = number of alleles; *H*_e_ = expected heterozygosity; *H*_o_ = observed heterozygosity; HWE = Hardy–Weinberg equilibrium probabilities; *n* = number of individuals sampled.

^a^See Appendix 1 for locality and voucher information for each population.

^b^Deviations from HWE were statistically significant at **P < 0.05 and ***P < 0.001. There were no values at *P* < 0.01. ns = not significant.

^‡^Unsuccessful amplification.

### Cross‐amplification in other species from *Filago*

Cross‐amplification was tested in nine additional species (Table 3) representing the three other subgenera recovered within *Filago* by Galbany‐Casals et al. (2010). Except for mf5, mf9, mf10, and mf14, all other loci were amplified (Table 3) for all species included in cross‐amplification. More specific PCR protocols could improve these results.

**Table 3:** Results of cross‐amplification of 10 polymorphic markers developed using *Filago gaditana* and *F. carpetana* within related *Filago* species.^a^

Species	Collector no.^b^^,^ c	mf5	mf8	mf9	mf10	mf13	mf14	mf19	mf25	mf26	mf28
***Filago*** **subg.** ***Filago***											
*F. albicans* Andrés‐Sánchez, M. M. Mart. Ort. & E. Rico (Clade G)	*SA202‐1*	150	200	75	—	175	—	260	290	320	330
	*SA202‐2*	150	200	75	—	175	—	260	290	320	330
	*SA202‐3*	150	200	75	—	175	—	+	290	320	330
*F. petro‐ianii* Rita & Dittrich (Clade H)	*SA249‐2*	100	200	75	—	200	200	260	290	300	330
	*SA249‐3*	100	200	75	—	200	200	260	290	300	330
	*SA249‐4*	—	—	—	—	—	—	—	—	—	—
*F. lusitanica* (Samp.) P. Silva (Clade H)	*SA1108‐1*	142	150	163	143	146–152	223	255	278	289–299	296
	*SA1108‐2*	142	150	163	143	146–152	223	255	278	289–299	296
	*SA1108‐3*	142	150	163	143	146–152	223	255	278	289–299	296
	*SA1108‐4*	142	150	163	143	146–152	223	255	278	289–299	296
	*SA1108‐5*	142	150	163	143	146–152	223	255	278	289–299	296
*F. ramosissima* Lange (Clade I)	*SA1090‐21*	150	200	75	—	175	200	260	290	300	330
	*SA1090‐22*	150	200	75	—	175	200	260	290	300	330
	*SA1090‐32*	150	200	—	—	175	+	260	290	300	330
*F. castroviejoi* Andrés‐Sánchez, D. Gut. Larr., E. Rico & M. M. Mart. Ort. (Clade F)	*SA1089‐14*	150	200	—	—	150–200	—	260	280	300	330
	*SA1089‐15*	150	200	—	75	150–200	—	260	280	300	330
	*SA1089‐16*	150	200	—	—	150–200	—	260	280	300	330
*F. germanica* (L.) Huds. (Clade D)	*MG‐1*	150	200	—	—	150–200	175	250	280	300	330
	*MG‐2*	150	200	—	75	150–175	175	250	280	300	330
	*MG‐3*	150	200	—	—	150–175	175	250	280	300	330
***Filago*** **subg.** ***Crocidion***											
*F. crocidion* (Pomel) Chrtek & Holub	*DG731‐17*	—	200	—	—	175	—	250	280	300	330
	*DG731‐18*	—	200	—	—	175	—	250	280	300	330
	*DG731‐19*	—	200	—	—	175	—	250	280	300	330
***Filago*** **subg.** ***Pseudevax***		—	200								
*F. hispanica* (Degen & Hervier ex Pau) Chrtek & Holub	*SA237‐1*	—	200	75	75	160	220	260	300	175–320	330
	*SA237‐2*	—	200	75	75	160	220	260	300	175–320	330
	*SA237‐3*	—	200	75	—	160	220	260	300	175–320	330
***Filago*** **subg.** ***Oglifa***		—									
*F. arvensis* L.	*BR128‐4*	—	160	—	—	160	—	260	—	—	—
	*BR128‐5*	—	160	—	—	160	—	260	290	—	330
	*BR128‐6*	—	160	—	—	160	—	260	290	320	330

Note

— = no amplification; + = successful amplification.

a Numbers shown represent the size in base pairs of the amplified fragments estimated by gel electrophoresis examination.

b See Appendix 1 for locality and voucher information for each collector number.

c DNA samples are deposited at Biobanco de ADN Vegetal, University of Salamanca, Salamanca, Spain. Specimens are deposited in the herbarium of the University of Salamanca (SALA; see Appendix 1).

## CONCLUSIONS

A set of polymorphic microsatellite markers for the genus *Filago* is reported here for the first time. Cross‐species amplification suggests that these markers may have utility for the entire genus. They will allow the development of phylogenetic, phylogeographic, and population genetic studies, which can contribute valuable information for species conservation, as well as data on reproductive systems.

## DATA ACCESSIBILITY

Sequence data for the 15 microsatellite loci were submitted to GenBank, and accession numbers are listed in Table 1. Sequence reads are available from the Dryad Digital Repository (https://doi.org/10.5061/dryad.94g0tc5; Gutiérrez‐Larruscain et al., 2018).

## Appendix 1 Voucher information for *Filago* samples used in this study.

**Table:**

Species	*n*	Herbarium code (Collector no.)^a^^,^ b	Locality	Geographic coordinates
*Filago lusitanica* (Samp.) P. Silva	5	SALA 157965 (*SA1108*)	Portugal: Terra de Miranda, Sequeiros	41°09′00.8″N, 07°04′04.7″W
*Filago gaditana* (Pau) Andrés‐Sánchez & Galbany	30	SALA 157396 (*SA865*)	Morocco: Gharb‐Chrarda‐Béni‐Hssn, Moulay Bousselham	34°52′51.2″N, 06°16′09.2″W
	27	SALA 158014 (*DP2044*)	Spain: Pontevedra, Isla de Arousa	42°31′55.1″N, 08°52′10.0″W
	28	SALA 158010 (*DP2040*)	Portugal: Setúbal, Santiago do Cacém	38°04′11.6″N, 08°47′01.5″W
	2	SALA139213 (*SA289* c)	Spain: Pontevedra, Isla de Arousa	42°31′55.8″N, 08°52′09.4″W
	3	SALA 139214 (*SA293* c)	Portugal: Minho, Esposense	41°12′26.2″N, 08°25′13.4″W
*Filago carpetana* (Lange) Chrtek & Holub	30	SALA 157952 (*SA1109*)	Spain: Salamanca, Masueco	41°13′55.2″N, 06°35′04.2″W
	27	SALA 162503 (*DG1052*)	Spain: Teruel, Frías de Albarracín	40°17′37.19″N, 01°35′50.3″W
	21	SALA 162522 (*SA1218*)	Spain: Burgos, Cubillo del Campo	42°08′37.3″N, 03°35′00.2″W
	3	SALA 110279 (*LD1059* c)	Spain: Zamora, Galende	42°07′15.0″N, 06°41′27.9″W
	3	SALA 134314 (*MO1804* c)	Spain: Salamanca, San Miguel de Valero	40°31′14.8″N, 05°54′23.7″W
*Filago arvensis* L.	3	SALA 110288 (*BR128*)	Macedonia: Mavrovo, Bistra Planina	41°43′12.3″N, 20°46′17.8″E
*Filago albicans* Andrés‐Sánchez, M. M. Mart. Ort. & E. Rico	3	SALA134823 (*SA202*)	Portugal: Alentejo, Ourique	37°41′03.3″N, 08°19′10.3″W
*Filago hispanica* (Degen & Hervier ex Pau) Chrtek & Holub	3	SALA139140 (*SA237*)	Morocco: Ifrane, Tizi‐n‐Tretten	33°25′43.3″N, 05°03′55.5″W
*Filago petro‐ianii* Rita & Dittrich	3	SALA 139206 (*SA249*)	Spain: Islas Baleares, Palma	39°33′58.9″N, 02°50′13.9″E
*Filago ramosissima* Lange	3	SALA 156143 (*SA1090*)	Spain: Almería, Tabernas	37°04′58.3″N, 02°19′07.2″W
*Filago crocidion* (Pomel) Chrtek & Holub	3	SALA 158953 (*DG731*)	Spain: Teruel, Frías de Albarracín	40°19′43.1″N, 01°41′26.9″W
*Filago castroviejoi* Andrés‐Sánchez, D. Gut. Larr., E. Rico & M. M. Mart. Ort.	3	SALA 156142 (*SA1089*)	Spain: Almería, Tabernas	37°04′58.3″N, 02°19′07.2″W
*Filago germanica* (L.) Huds.	3	SALA 160405 (*MG*)	Spain: Girona, Roses	42°17′03.11″N, 03°10′53.05″W

Note

*n* = number of individuals sampled.

^a^Herbarium specimens are deposited at the herbarium of the University of Salamanca (SALA), Salamanca, Spain.

^b^Abbreviations (collector no.): BR = Blanca Rojas‐Andrés; DG = David Gutiérrez‐Larruscain; DP = Daniel Pinto Carrasco; LD = Luis Delgado; MG = Merçe Galbany; MO = M. Montserrat Martínez‐Ortega; SA = Santiago Andrés‐Sánchez.

^c^Specimens used for the preparation of the microsatellite library.

## Appendix 2 Primers rejected during the study and reasons for discarding.

**Table:**

Locus	Primer sequences (5′–3′)	Repeat motif	PCR product size	*T*_a_ (°C)	Reason for discarding
mf2	F: GGCCTAGCTAGCAGATCCC	(AAG)_6_	120	52–53	Unsuccessful amplification
	R: TCTTCTCCGTCACGCCTC				
mf4	F: GGCCTAGCTAGCAGAATCCA	(ACC)_5_	121	52–53	Unsuccessful amplification
	R: CCACCTGACGACCCACTAAT				
mf6	F: GGCCTAGCTAGCAGAATCAA	(ACTCCT)_5_	129	52–53	Unsuccessful amplification
	R: TCCAGAAGTCTATCATCGTTATTG				
mf11	F: GCTAGCAGAATCTCGGTTGG	(ACC)_5_	142	52–53	Unsuccessful amplification
	R: AGGAGGAACATCAATCCTCG				
mf15	F: AGGCATTGTTAGGGTTGGTG	(ACC)_5_	148	52–53	Unsuccessful amplification
	R: CAAACATTCCTGGATATGGGA				
mf16	F: GGCCTAGCTAGCAGAATCCA	(AAC)_5_	206	52–53	Unsuccessful amplification
	R: TCCTGTAACCGGCATTCCT				
mf17	F: GCCTAGCTAGCAGAATCCGA	(AAC)_7_	208	52–53	Unsuccessful amplification
	R: TGGTAAGGGTCTTCCTCATACAA				
mf18	F: AGGCCTAGCTAGCAGAATCAA	(AAATG)_6_	231	52–53	Unsuccessful amplification
	R: AAGGTGTTACCACTAGTCAGCTTG				
mf21	F: ACCCGAATGCATCAGGTAAC	(AGC)_5_	240	52–53	Unsuccessful amplification
	R: CCCGAGATTTCTCAACGTCT				
mf22	F: CACGTTGCAGCTAGCGTTAT	(AGG)_6_	253	52–53	Unsuccessful amplification
	R: CGATACACATGGAGCACGTC				
mf23	F: GGCCTAGCTAGCAGAATCTACC	(AAC)_5_	257	52–53	Unsuccessful amplification
	R: GGTTTGGGTGAGTTGAGCAT				
mf24	F: AAGGCCTAGCTAGCAGAATCAA	(AAC)_5_	260	52–53	Unsuccessful amplification
	R: TGAGCAAGATTAGAAGTACCCTCA				
mf27	F: GTTTAAGGCCTAGCTAGCAGAA	(AAG)_6_	280	52–53	Unsuccessful amplification
	R: TGGTGGTTATAACGGAGAATGG				
mf29	F: CACCATCCTTTCAAACACCC	(AAC)_6_	281	52–53	Unsuccessful amplification
	R: AAGCTTCCTGAAGGCGAAA				
mf30	F: AAGGCCTAGCTAGCAGAATCTC	(AAC)_8_	406	52–53	Unsuccessful amplification
	R: GTGGTCGGTTGCTCGTTATC				

Note

*T*_a_ = annealing temperature.
